# Nested Dilation Networks for Brain Tumor Segmentation Based on Magnetic Resonance Imaging

**DOI:** 10.3389/fnins.2019.00285

**Published:** 2019-04-05

**Authors:** Liansheng Wang, Shuxin Wang, Rongzhen Chen, Xiaobo Qu, Yiping Chen, Shaohui Huang, Changhua Liu

**Affiliations:** ^1^Fujian Key Laboratory of Sensing and Computing for Smart City, School of Information Science and Engineering, Xiamen University, Xiamen, China; ^2^Department of Computer Science, School of Information Science and Engineering, Xiamen University, Xiamen, China; ^3^Department of Electronic Science, Fujian Provincial Key Laboratory of Plasma and Magnetic Resonance, School of Electronic Science and Engineering (National Model Microelectronics College), Xiamen University, Xiamen, China; ^4^Department of Medical Imaging, Chenggong Hospital Affiliated to Xiamen University, Xiamen, China

**Keywords:** brain tumor segmentation, nested dilation networks, residual blocks nested with dilations, squeeze-and-excitation blocks, coarse-to-fine

## Abstract

**Aim:** Brain tumors are among the most fatal cancers worldwide. Diagnosing and manually segmenting tumors are time-consuming clinical tasks, and success strongly depends on the doctor's experience. Automatic quantitative analysis and accurate segmentation of brain tumors are greatly needed for cancer diagnosis.

**Methods:**This paper presents an advanced three-dimensional multimodal segmentation algorithm called nested dilation networks (NDNs). It is inspired by the U-Net architecture, a convolutional neural network (CNN) developed for biomedical image segmentation and is modified to achieve better performance for brain tumor segmentation. Thus, we propose residual blocks nested with dilations (RnD) in the encoding part to enrich the low-level features and use squeeze-and-excitation (SE) blocks in both the encoding and decoding parts to boost significant features. To prove the reliability of the network structure, we compare our results with those of the standard U-Net and its transmutation networks. Different loss functions are considered to cope with class imbalance problems to maximize the brain tumor segmentation results. A cascade training strategy is employed to run NDNs for coarse-to-fine tumor segmentation. This strategy decomposes the multiclass segmentation problem into three binary segmentation problems and trains each task sequentially. Various augmentation techniques are utilized to increase the diversity of the data to avoid overfitting.

**Results:** This approach achieves Dice similarity scores of 0.6652, 0.5880, and 0.6682 for edema, non-enhancing tumors, and enhancing tumors, respectively, in which the Dice loss is used for single-pass training. After cascade training, the Dice similarity scores rise to 0.7043, 0.5889, and 0.7206, respectively.

**Conclusion:** Experiments show that the proposed deep learning algorithm outperforms other U-Net transmutation networks for brain tumor segmentation. Moreover, applying cascade training to NDNs facilitates better performance than other methods. The findings of this study provide considerable insight into the automatic and accurate segmentation of brain tumors.

## 1. Introduction

Brain tumors are one of the deadliest cancers worldwide. Gliomas are the most common primary craniocerebral tumor and are caused by the carcinogenesis of glial cells in the brain and spinal cord (Bauer et al., 2013). In pathology, gliomas can be classified as low-grade or high-grade according to the malignant degree of the tumor cells (Cho and Park, 2017; Wang et al., 2018b). Low-grade gliomas are mainly represented by low-speed cell division and proliferation, whereas high-level gliomas are characterized by rapid cell division and proliferation accompanied by angiogenesis, hypoxia, and necrosis (Gerlee and Nelander, 2012; Bogdańska et al., 2017). Although significant advances have been made in healthcare so far, the vast majority of gliomas are incurable, except for a small number of low-grade gliomas, which can be completely resected surgically. Gliomas can be further divided into different tumor sub-regions according to the severity of the tumor cells, such as edemas, non-enhancing tumors, and enhancing tumors. Magnetic resonance imaging (MRI) is the most frequently used and most effective noninvasive auxiliary diagnostic tool (Wen et al., 2010; Yang et al., 2018), providing a reference for the formulation of treatment programs (Mazzara et al., 2004). Brain tumors are usually imaged with different MRI modalities, and these images are interpreted by image analysis methods (Bauer et al., 2013). The MRI sequence usually includes four different modalities: T1-weighted, T2-weighted, post-contrast T1-weighted, and fluid-attenuated inversion-recovery (FLAIR). Different MRI modalities are employed for different diagnosis tasks in clinical diagnosis and treatment. However, it is still a daunting task for clinicians to diagnose diseases with MRI, because there is a wide variation in the size, shape, regularity, location, and heterogeneous appearance of brain tumors (Dong et al., 2017). Therefore, automatic quantitative analysis and accurate segmentation of brain tumors are greatly needed clinically to help doctors make accurate diagnoses.

CNNs have become a prominent deep learning method and have been used to make a series of breakthroughs in different tasks, including computer vision (Krizhevsky et al., 2012; Long et al., 2015; Ren et al., 2015). The success of CNNs is credited to their ability to independently learn deep features instead of relying on manual features. With historical opportunities provided by a strong calculation capability and large numbers of annotations, the development of CNNs has been explosive. The original LeNet5 (LeCun et al., 1998) was proposed in 1998 with five layers, establishing the modern structure of CNNs. Krizhevsky et al. (2012) presented a classical CNN structure called “AlexNet, and made a historic breakthrough. The great success of AlexNet stimulated new research on CNNs. ZFNet (Zeiler and Fergus, 2014), VGGNet (Simonyan and Zisserman, 2014), GoogLeNet (Szegedy et al., 2015), and ResNet (He et al., 2016) were successively presented with more layers and better performances. Huang et al. (2017) used a more radical dense connection mechanism to maximize the flow of information. Hu et al. (2017) proposed an SE network that modeled the interdependencies between feature channels, adaptively learning important information. All of these CNN studies made it possible to apply neural networks to medical image processing.

Recent reports have shown that CNNs outperform state-of-the-art medical image analyses (Li et al., 2017; Lin et al., 2018). MRI-based brain tumor segmentation is a task that still requires extensive attention. Extant methods for automatic brain tumor segmentation are diverse. DeepMedic (Kamnitsas et al., 2016b) was designed as a dual-pathway three-dimensional (3D) network with 11 layers, to simultaneously process images at different scales and combine the results with fully connected layers. Kamnitsas et al. (2016a) and Castillo et al. (2017) further improved the architecture of DeepMedic by adding residual connections and parallel pathways. U-Net (Ronneberger et al., 2015) was proposed to train an end-to-end network with few images for the accurate segmentation of biomedical images. Many architectures similar to U-Net have been widely adopted for brain tumor segmentation. Kayalibay et al. (2017) and Isensee et al. (2017) employed deep supervision by combining segmentation layers from different levels in the localization pathway. Iqbal et al. (2018) increased the number of U-Net layers and trained the network with the Dice loss. Le and Pham (2018) used the U-Net architecture to extract features and put them into an ExtraTrees classifier. Zhao et al. (2018) integrated fully convolutional neural network (FCNN) and conditional random field (CRF) and trained three models using two-dimensional (2D) image patches obtained from axial, coronal, and sagittal views. A voting-based fusion strategy was used to obtain segmentation results. To deal with the class imbalance problem, Wang et al. (2017) proposed a triple-cascaded framework for brain tumor segmentation. Three similar networks were used to segment the entire tumor (all lesions, including edema, non-enhancing tumors, and enhancing tumors), and the tumor core (all lesions except edema). They then sequentially enhanced tumor core. Zhou et al. (2018) drew upon lesions with coarse-to-fine medical image segmentation methods and proposed a single multitask CNN that could learn correlations between different categories. Partial model parameters can be shared when different tasks are being trained according to different sets of training data to utilize the underlying correlation among classes.

We propose a CNN-based 3D segmentation algorithm, the NDN, which can handle multimodal images. Instead of simple convolution layers, residual blocks are stacked in the U-Net architecture to simplify optimization. The SE blocks used in NDNs fuse the global information and adaptively learn important information from each channel. A new block i.e., residual blocks nested with dilations (RnD) enlarges the receptive fields and avoids the gridding effect. RnD blocks can enrich information in shallow layers by using dilation convolutions while retaining detailed information during the rapid expansion of receptive fields by using residual connections. The cascade training strategy is adopted to train three tasks individually to deal with the class imbalance problem.

## 2. Materials and Methods

This section describes the proposed NDNs algorithm for detailed brain tumor segmentation, including the data preprocessing, network architecture, training strategy, and post-processing methods. We also concisely describe the experimental design.

### 2.1. Data Acquisition and Preprocessing

#### 2.1.1. Data Acquisition

Most of the data used in this work are downloaded from the Medical Segmentation Decathlon (MSD) organized by the 21st Annual Conference on Medical Image Computing and Computer-Assisted Intervention (MICCAI) 2018. A small number of low-grade glioma data are abstained from MICCAIs Multimodal Brain Tumor Segmentation (BraTS) Challenge of the same year. These are used to test the stability of the proposed algorithm. The images for each patient comprise four scanning sequences: T1-weighted, T2-weighted, post-contrast T1-weighted, and FLAIR. Every scan is aligned to the same anatomical template space and interpolated into 1 × 1 × 1*mm*^3^ with an image size of 240 × 240 × 155 voxels. The purpose of the study is to segment brain tumors (i.e., gliomas) into three different classes: edemas, non-enhancing tumors, and enhancing tumors. All data are labeled and verified by an expert human rater. Efforts were made to mimic the accuracy required for clinical use.

#### 2.1.2. Data Preprocessing and Augmentation

**Figure 1:**
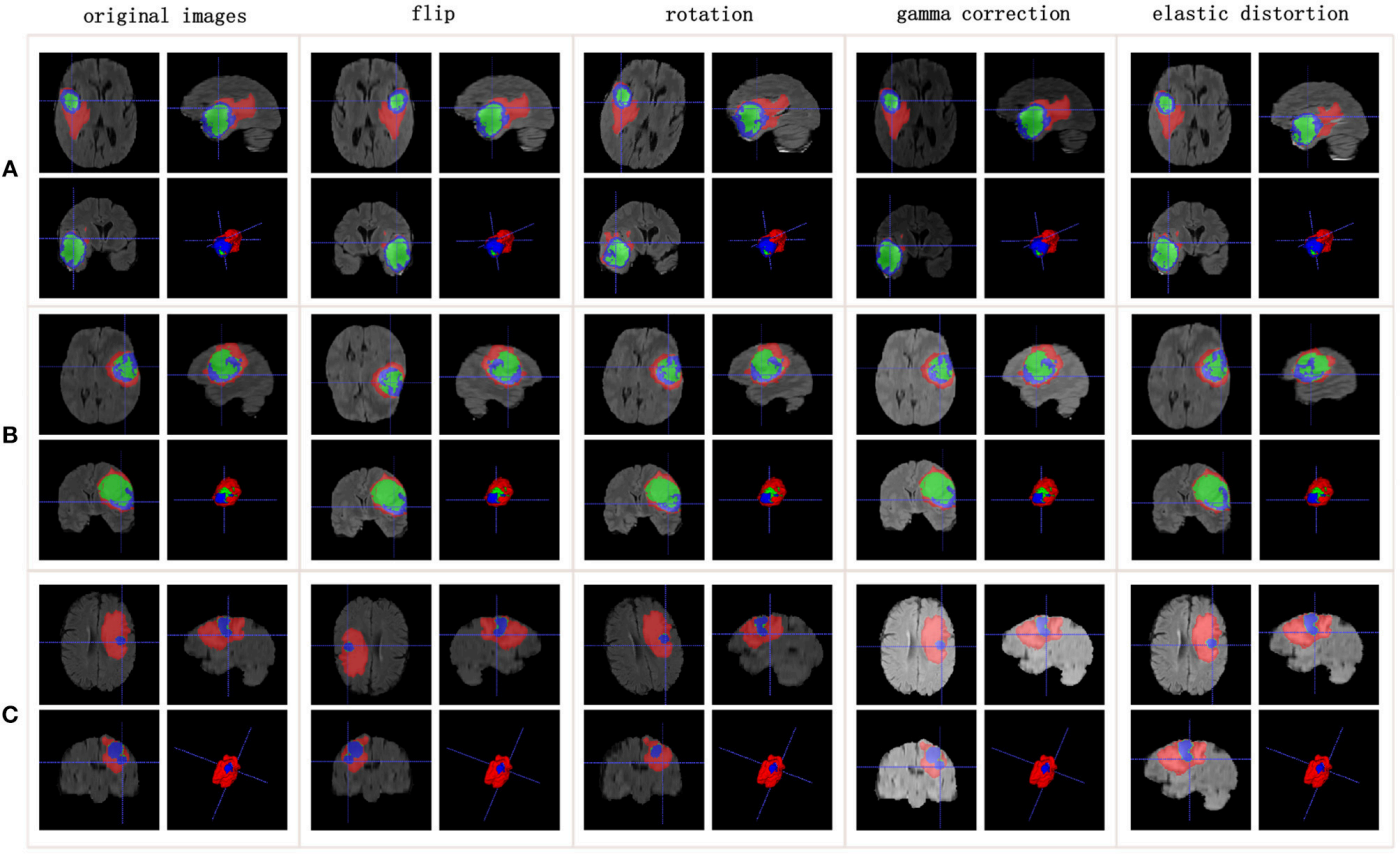
Data augmentation results. Rows list three samples for different patients. Columns represent different data augmentation operations. **(A-C)** list three samples for different patients.

Training an effective neural network requires thousands or even tens of thousands of data. However, the quantity of available medical images is usually well short of that. To avoid overfitting, more training data need to be generated from the limited images and annotations. Our method applies the following data augmentation techniques to make reasonable changes to the image shapes: flip the x-, y-, or z-axis with a probability of 50%; rotate the images with a rotation angle of −15° to 15°; apply gamma correction with the gamma value varied randomly from 0.4 to 1.6; and apply elastic distortion. Figure 1 shows the data augmentation.

Images from multiple modalities may have varying intensity ranges. When the intensity values are not standardized, it is detrimental to the training of the neural network. Normalization is critical to allow images from different modalities to be trained with one algorithm. In our study, each modality is normalized individually by subtracting the mean from the value for each patient and dividing it by the standard deviation. The useless black borders in the images along the x- and y-axes are also removed. On the z-axis, we note that the head and tail of the image slices are uninformative. Therefore, 70% of the slices used for the network input are captured from the middle.

### 2.2. Residual Blocks

**Figure 2:**
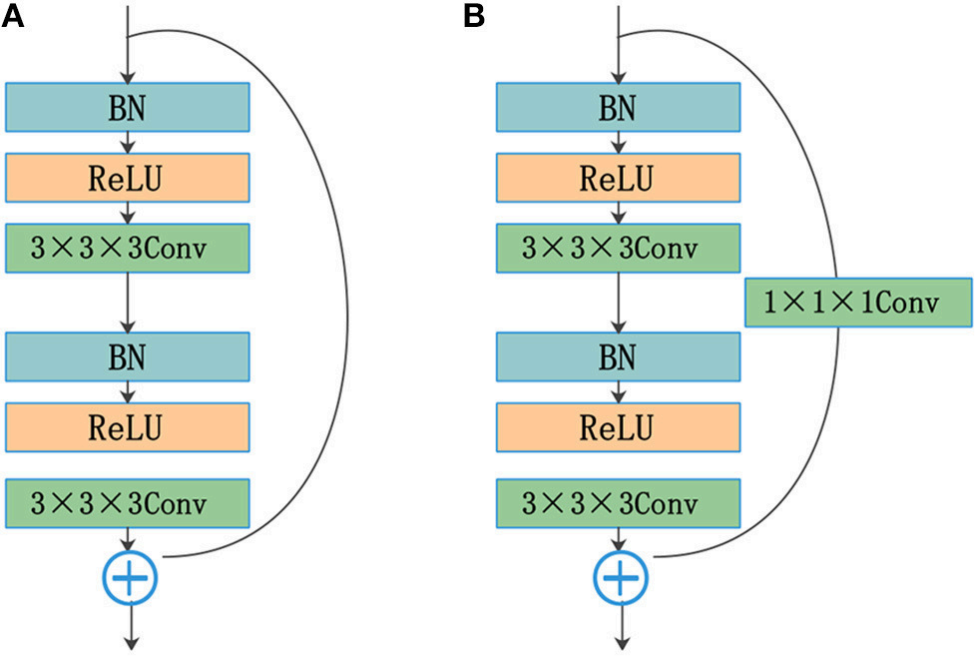
Residual blocks: **(A)** shortcut connection and **(B)** shortcut connection with the convolution layer.

He et al. (2016) reformulated the layers as residual blocks and yielded unusually brilliant results in the 2015 ImageNet competition. Instead of simply stacking convolution layers to fit a desired underlying mapping, they added identity mapping, which was easier to optimize. The residual blocks depicted in Figure 2A are achieved by a shortcut connection and element-wise addition operation, performed on the input and output feature maps of the blocks, channel-by-channel. The operating principle of the residual blocks can be defined as

(1) $$\begin{array}{l} y=F\left(x,W_{i}\right)+x, \end{array}$$

where *x* and *y* are the input and output vectors of the relevant layers; and *F*(*x, W*_*i*_) is the mapping function for the residual path. The results of *F*(*x, W*_*i*_) should have the same dimensions as *x*. Otherwise, we can perform linear mapping on the shortcut connection (Figure 2B). This simple algorithm does not add additional parameters or computations to the network, but it greatly increases the training speed of the model and improves the training effect.

The standard convolutional layers of a U-Net are replaced by the residual structure shown in Figure 2A. The residual path comprises two convolution layers with a kernel size of 3, followed by a batch normalization (BN) operation (Ioffe and Szegedy, 2015) and a rectified linear unit (ReLU). The input and output of the residual path are added element by element. The results of the residual blocks are fed directly into subsequent network layers.

### 2.3. SE Blocks

**Figure 3:**
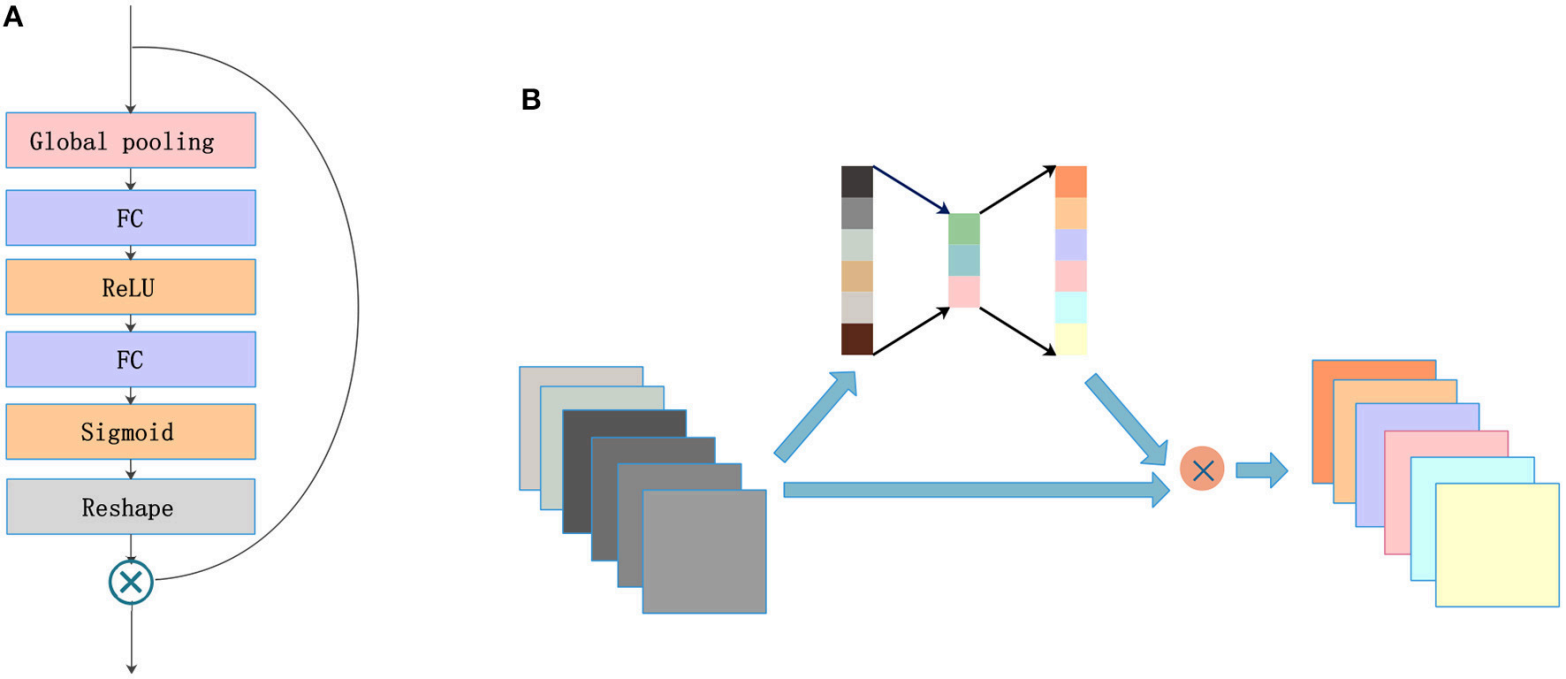
SE blocks: **(A)** architecture and **(B)** concept map.

A lot of research has recently been accomplished to strengthen the learning power of CNNs and to improve their performance. Hu et al. (2017) introduced the SE blocks to enhance the representations of features produced by a convolutional network. SE blocks embed the global spatial information into the channel vector by encoding each channel dependency with a fully connected operation. It allows the network to pay different amounts of attention to each channel according to the importance of the feature maps. Figure 3A illustrates the structure of SE blocks. The features are first passed through a squeeze operation achieved by a global average pooling layer to aggregate global information per channel for the whole image. Then, the outputs are fed into an excitation operation to get the final weights for each channel. The excitation operation is achieved by using two fully connected layers: one with ReLU activation and another with a sigmoid. Finally, the weight vectors are reshaped to (1, 1, 1, *C*), where *C* is the number of the feature maps and are applied to each feature map by the multiply operation. The SE blocks emphasize useful features and suppress useless features through weights like an attention mechanism.

SE blocks have a simple structure and can be used directly in existing state-of-the-art architectures. We draw on experience with the attention mechanism and nested SE blocks in the architecture to help the network focus on important feature maps. As shown in Figure 3B, feature maps with size (*X, Y, Z, C*) are put into SE blocks. Then, the blocks generate a significant coefficient for each channel, finally gaining outputs with different weights and the same size as the inputs.

### 2.4. RnD Blocks

The traditional up-sampling and down-sampling structures lead to a loss of internal structure, and the information of small objects cannot be reconstructed. To solve this problem, Yu and Koltun (2015) presented a model with dilated convolutions that can increase the receptive fields without reducing the resolution or increasing the parameters. Chen et al. (2014, 2017, 2018) used dilated convolutions in their networks and achieved good performance for dense prediction tasks. However, standard dilated convolution causes a gridding issue that will harm small objects. Wang et al. (2018a) proposed a hybrid dilated convolution (HDC) framework, which can not only expand receptive fields but also mitigate the gridding issue. Implementing the HDC framework requires two conditions to be met. First, the dilation rates of a groups dilated convolutions should not have a common divisor > 1. The maximum distance between two nonzero values is defined as follows:

(2) $$\begin{array}{l} M_{i}=max\left[M_{i+1}-2r_{i},M_{i+1}-2\left(M_{i+1}-r_{i}\right),r_{i}\right], \end{array}$$

where *r*_*i*_ is the dilation rate in layer *i*, and *M*_*i*_ is the maximum dilation rate from layer 0 to layer *i*. The second condition requires satisfying *M*_*i*_ < *K*, where *K* is the kernel size.

**Figure 4:**
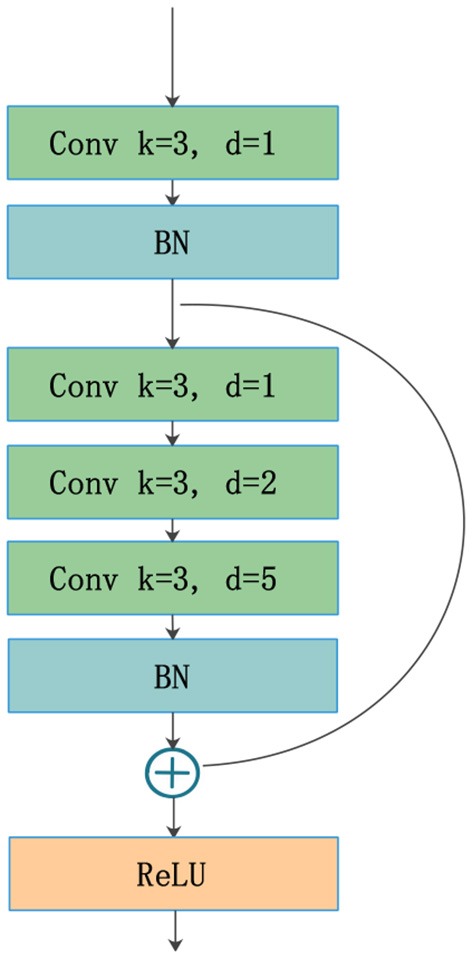
Architecture of RnD blocks with different dilation rates.

The standard U-Net architecture does not get enough semantic information in the shallow layers because of the limited receptive fields. This is harmful to feature fusion in the first few cross-layer connections. To resolve this issue and avoid the gridding effect, we draw on an idea from the HDC framework. RnD blocks (Figure 4) are built to enlarge receptive fields in the first two layers of the network. This new type of block can obtain more extensive local information via 3 convolution layers with different dilation rates (e.g., 1, 2, 5). The kernel size is 3 for all dilated convolutions, which are followed by a ReLU activation. The residual connection in RnD blocks helps retain information and fill details during the rapid expansion of receptive fields.

### 2.5. NDNs

**Figure 5:**
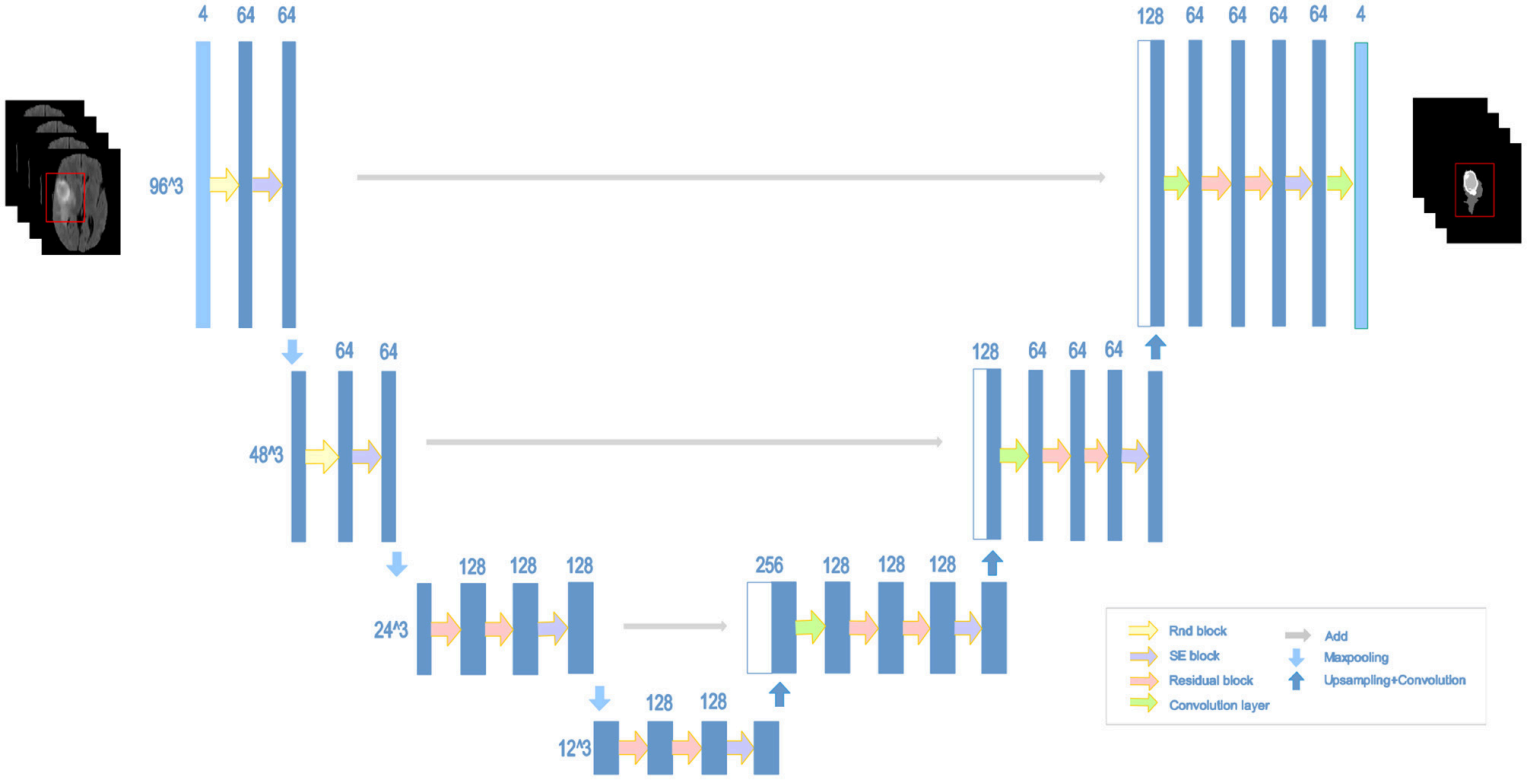
Architecture of the proposed NDNs with SE blocks and RnD blocks.

The structure of our proposed NDNs is shown in Figure 5. The architecture is inspired by U-Net, which is a stable encoder–decoder network designed for limited data training, especially for medical images. Here, we carefully modify the standard U-Net to make it perform better for the brain tumor segmentation task. First, we use 3D convolution layers rather than 2D to adapt images from multiple modalities. The classic encoder–decoder structure that fuses the lower features in the shallow layers and higher features in the deep layers is retained to ensure the stability of the proposed network. The architecture comprises three max-pooling layers to capture context and three up-sampling layers to enable precise localization. To obtain enough receptive fields, the first two encoder modules adopt RnD blocks to enrich the low-level features. This is followed by an SE block and a max-pooling layer. In the decoder part, each module comprises a stack of residual blocks, an SE block, and an up-sampling layer. The BN is employed immediately after each convolution and before activation. As shown in Figure 5, the network can obtain rich information to boost essential features and achieve a stable effect.

### 2.6. Cascade Training

The cascade strategy trains different models for each category sequentially, showing ideal results. Coarse-to-fine medical image segmentation is becoming increasingly popular because of the class imbalance problem. Cascaded models decompose complex problems into simple ones and capitalize on the hierarchical structure of tumor sub-regions. A single model is trained repeatedly to segment substructures of brain tumors hierarchically and sequentially. Each sequence is handled as a binary segmentation problem. The first task is to segment the entire tumor including edemas, enhancing tumors, and non-enhancing tumors. These three classes are regarded as a binary segmentation problem. Then, NDNs are trained to crop the target. After the first stage of training, the entire tumor region is segmented in the 3D volumes of a patient. A cuboid sub-region, based on the entire tumor, is used as inputs to the network to segment the enhancing and non-enhancing tumors together. Similarly, the third training differentiates enhancing tumors from non-enhancing ones by using the cuboid sub-region produced by the second stage as input.

**Figure 6:**
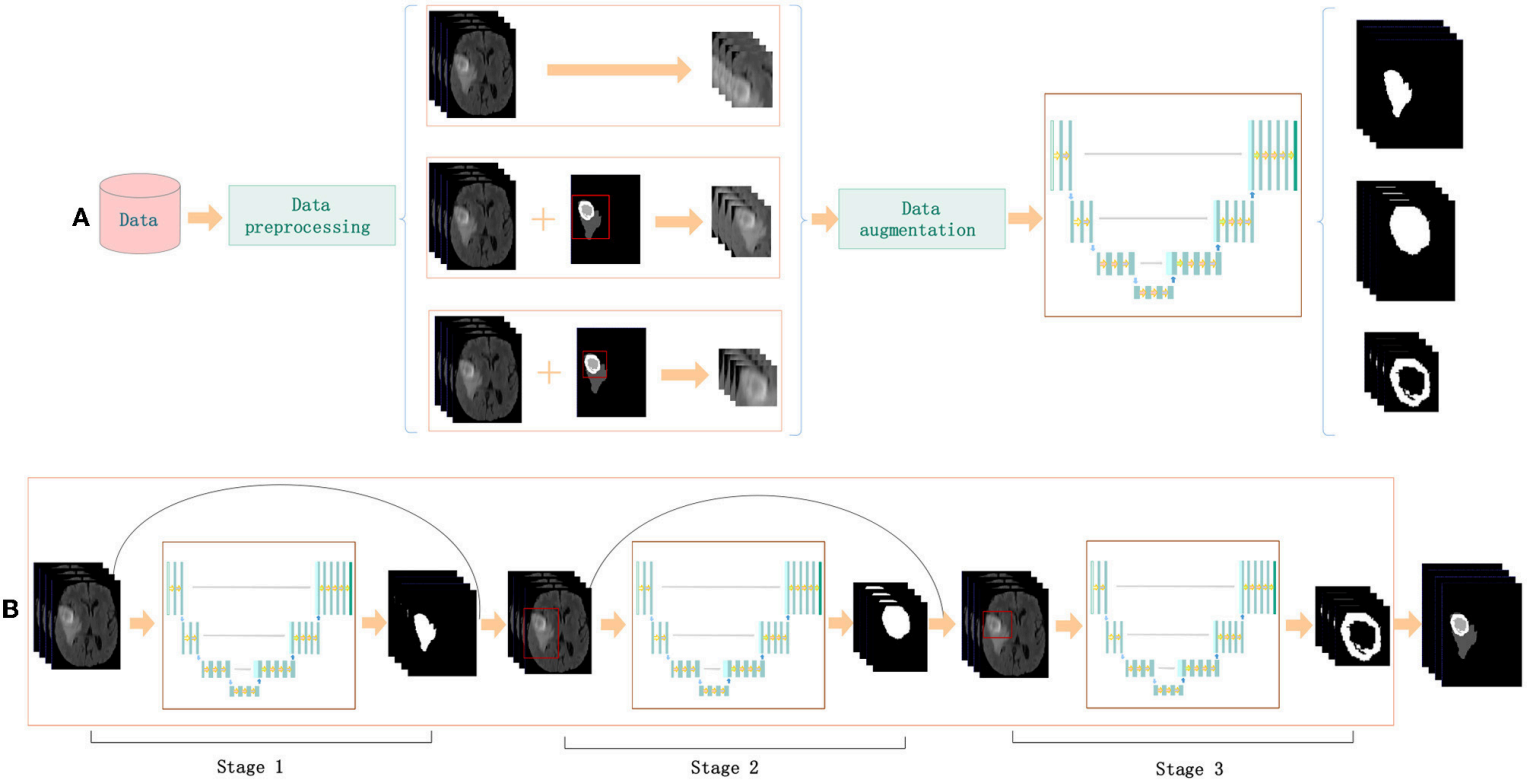
Cascade strategy applied to NDNs: **(A)** training and **(B)** testing.

In the training, the input of the network is generated based on the ground truth, as shown in Figure 6A. In the testing, the results of the previous stage are extended by 32 pixels on the x- and y-axes, and 8 slices on the z-axis as the input for the next stage. The process is described in Figure 6B. Finally, we integrate the three binary segmentation tasks to obtain the final segmentation results of multiple classes. Cascade training offers a way to adaptively alleviate the class imbalance problem of brain tumor segmentation.

### 2.7. Post-processing

Post-processing is further used to improve the segmentation results of NDNs. During data processing, we noticed that the brain tumors for all patients in the 3D volumes were of a single connected domain. Thus, isolated small clusters should be removed from the results. More specifically, connected domain analysis should be performed to retain the maximal region and remove other smaller clusters to better fit the ground truth. Moreover, some patients are observed to have benign tumors, which means that the gliomas only comprise edemas and non-enhancing tumors. Some small clusters are erroneously classified as enhancing tumors in our task instead of benign tumors, which harms the segmentation results. To deal with this issue, we impose volumetric constraints by removing enhancing tumor clusters in the segmentation that are smaller than a predefined threshold.

### 2.8. Dice Similarity Score

In our work, the Dice similarity score is calculated for quantitative evaluation. This performance metric measures the similarity between the ground truth and predicted results. The Dice similarity score is defined as follows:

(3) $$\begin{array}{l} DSC=\frac{2TP}{\left(FP+2TP+FN\right)}, \end{array}$$

where *TP*, *FP*, and *FN* are the numbers of true positives, false positives, and false negatives, respectively.

MSD and BraTS 2018 provide three different tumor regions that can be described as edemas, enhancing tumors, and non-enhancing tumors. The Dice similarity scores are calculated for each tumor region to evaluate the segmentation results, and the scores are compared with those of other methods.

### 2.9. Experimental Design

We conduct three groups of experiments according to different requirements, which we describe in this section.

Experiment 1: We explored the effects of different network structures on brain tumor segmentation. Ronneberger et al. (2015) developed a U-Net architecture based on the fully convolutional network (FCN) (Long et al., 2015), which can work with very few training images and yield more precise segmentation. Some new architectures derived from U-Net have appeared and have been applied to the field of medical image processing. In Experiment 1, the standard Conv + BN + ReLU module in U-Net was replaced by frequently used blocks, such as residual blocks and dense blocks separately for comparison with the proposed NDNs.

Experiment 2: Different loss functions were attempted with NDNs to improve segmentation results. The loss function quantifies the amount by which the predicted value deviates from the actual value. Choosing a suitable loss function benefits both the training process and the results of brain tumor segmentation. In Experiment 2, different loss functions were applied to the brain tumor segmentation task: the categorical cross-entropy loss, Dice loss, and focal loss. The Dice similarity scores are calculated for each task.

Experiment 3: The proposed method was compared with other state-of-the-art methods. We implemented several previously published algorithms and trained the networks with the same datasets. The brain tumors comprise of edemas, enhancing tumors, and non-enhancing tumors with very different volumes, resulting in an imbalanced number of samples in each class. This category imbalance problem impairs the performance of a deep network. In Experiment 3, a cascade strategy was used to train NDNs, which decomposed a multiple classification problem into multiple binary classification problems. The segmentation results of the cascaded NDNs were compared with several state-of-the-art methods according to the Dice similarity score.

### 2.10. Implementation Details

All networks were implemented in Keras (Chollet et al., 2015) 2.1.2 using the Tensorflow (Abadi et al., 2016) 1.4.0 backend. Adaptive moment estimation (Kingma and Ba, 2014) was used as an optimizer with an initial learning rate of 0.0001, a momentum of 0.9, and a weight decay of 0.00001. Training was implemented on an NVIDIA 1080 Ti GPU with a version of CUDA 8.0 for 300 epochs. We did not use a dropout (Hinton et al., 2012) but rather L2 regularization and BN for the whole network structure. We cropped 96 × 96 × 48 patches as inputs close to the ground truth from images and annotations. All networks were trained from scratch with a batch size of 4.

## 3. Experiments and Results

In this section, we explain the advantages of the proposed algorithm with regard to brain tumor segmentation. The Dice similarity score is adopted as the evaluation criterion for each model. Edemas, non-enhancing tumors, and enhancing tumors were trained together with single NDNs in Experiments 1 and 2 for the sake of fairness. In Experiment 3, however, the cascade training strategy was used to train NDNs for each class, which was then compared with the state-of-the-art methods.

### 3.1. Experiment 1

**Figure 7:**
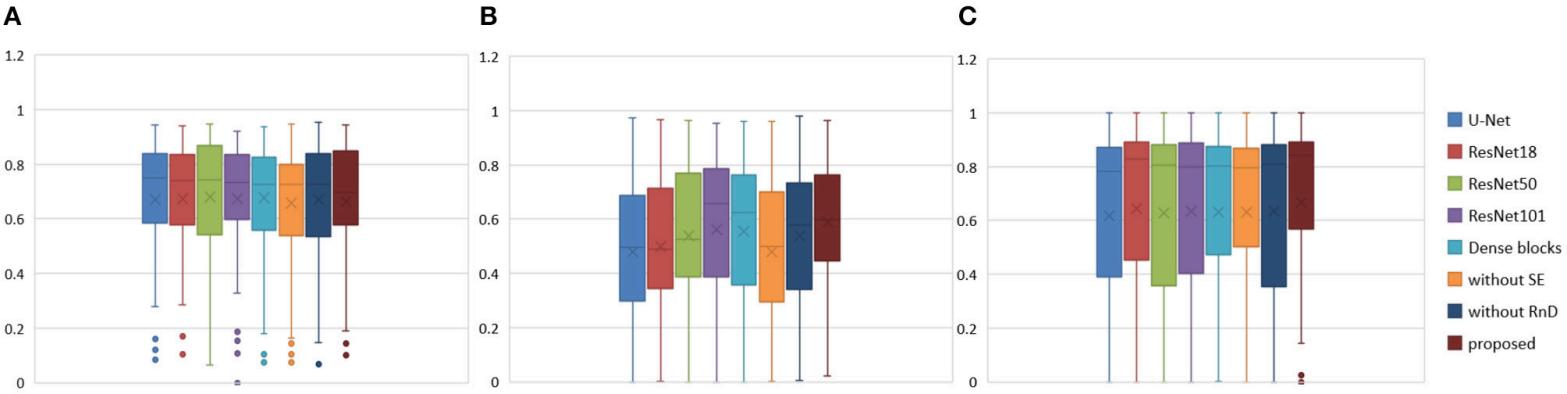
Boxplots for each method in Table 1. Dice similarity scores for **(A)** edema, **(B)** non-enhancing tumors, and **(C)** enhancing tumors. The symbol “ × ” marks the mean.

To prove the effectiveness of the NDNs structure, different U-Net-like networks were trained with the same brain tumor dataset for comparison. A traditional 3D U-Net with three down-sampling layers and three symmetric up-sampling layers was trained first. It consisted of two convolution layers used repeatedly with a kernel size of 3, similar to the standard 2D U-Net structure presented by Ronneberger et al. (2015). The filter number was doubled at the end of each down-sampling layer and halved after each up-sampling layer. Then, the repeated convolution layers were replaced by residual blocks and dense blocks to be trained. ResNet18, ResNet50, and ResNet101 were each employed as an encoder path, and the decoder path was consistent with the expanding path in 3D U-Net. For the dense U-Net, dense blocks were used as substitutes for the two repeated convolution layers, and each dense block had four dense connected convolution layers. Finally, we studied the effect of the NDNs architecture with SE blocks or RnD blocks only. Table 1 lists the Dice similarity scores calculated for brain tumor segmentation with these networks, and Figure 7 presents the boxplots for each class. Note that all networks were trained with the Dice loss in Experiment 1.

**Table 1:** Comparison of different U-Net-like architectures: (A) standard 3D U-Net; (B) U-Net with residual blocks; (C) U-Net with dense blocks; (D) NDNs without SE blocks; (E) NDNs without RnD blocks; and (F) NDNs network.

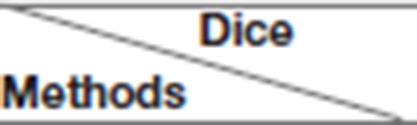		**Edema**	**Non-enhancing tumor**	**Enhancing tumor**
A		0.6686	0.4734	0.6169
B	ResNet18	0.6645	0.5022	0.6455
	ResNet50	**0.6792**	0.5314	0.6289
	ResNet101	0.6752	0.5617	0.6342
C		0.6734	0.5527	0.6287
D		0.6590	0.5612	0.6305
E		0.6725	0.536	0.638
F		0.6652	**0.5880**	**0.6682**

*The bold values mean the best result (the highest dice value) for each class*.

**Figure 8:**
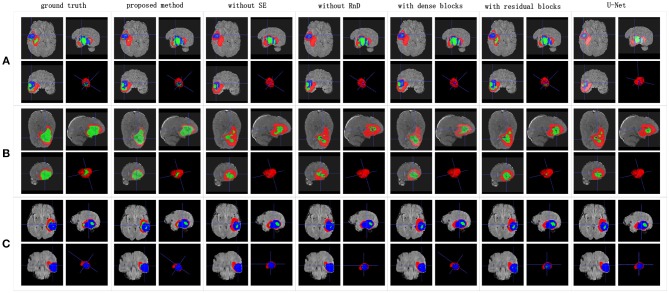
Brain tumor segmentation results predicted by different U-Net-like networks. The rows represent three samples from different patients, and the columns represent results predicted by each U-Net-like network. The organizers provided the ground truth images. **(A-C)** list three samples for different patients.

We achieved better results for non-enhancing tumor segmentation and enhancing tumor segmentation with NDNs than with the other U-Net-like architectures. According to Table 1, the non-enhancing tumor results segmented by NDNs are about 2.6% better than U-Net with ResNet101, and the enhancing tumor segmentation results are at least 2.0% better than the other methods. However, the proposed algorithm lacked the ability to segment the edema part with a result of 0.6652, which is worse than the other U-Net-like algorithms. Figure 8 presents the ground truth and prediction results for different U-Net-like architectures from different perspectives.

### 3.2. Experiment 2

Class imbalance is a severe issue in medical image segmentation and needs to be carefully tackled. The data provided by MSD and BraTS 2018 are heavily imbalanced, especially the classes of the non-enhancing tumors and enhancing tumors. To alleviate the class imbalance, we use a Dice loss function. We also explore the effects of other loss functions on NDNs for comparison. The categorical cross-entropy is used as a base loss function:

(4) $$\begin{array}{l} Crossentropy\left(p,q\right)=-\frac{1}{N}\underset{x,y,z}{\sum }\underset{k}{\sum }p_{x,y,z}^{k}\log q_{x,y,z}^{k}, \end{array}$$

where $p_{x,y,z}^{k}$ and $q_{x,y,z}^{k}$ correspond to the ground truth and predicted results for class *k*, and *N* is the total number of samples. Based on previous experience, the class imbalance can be addressed by associating different weights with individual classes. Therefore, the weighted categorical cross-entropy is also used:

(5) $$\begin{array}{l} W\text{\_}Crossentropy\left(p,q\right)=-\frac{1}{N}\underset{x,y,z}{\sum }\underset{k}{\sum }w^{k}p_{x,y,z}^{k}\log q_{x,y,z}^{k}, \end{array}$$

where *w*^*k*^ is the weight for class *k*. Here the weights for the background, edema, non-enhancing tumors, and enhancing tumors are defined as (1, 1, 2, 1) respectively. The focal loss function described by Lin et al. (2017) for dense object detection is a modified version of binary cross-entropy and is aimed toward low-confidence labels. We adopt a multiclass focal loss for the segmentation task:

(6) $$\begin{array}{l} Focal\left(p,q\right)=-\frac{\sum_{x,y,z}\sum_{k}p_{x,y,z}^{k}{\left(1-q_{x,y,z}^{k}\right)}^{\gamma }\log q_{x,y,z}^{k}}{\sum_{x,y,z}\sum_{k}p_{x,y,z}^{k}}, \end{array}$$

where ${\left(1-q_{x,y,z}^{k}\right)}^{\gamma }$ is a modulating factor and the value of γ is set to 2.0 in our algorithm. Finally, our proposed model is trained with the following Dice loss to segment different parts of the brain tumors:

(7) $$\begin{array}{l} Dice\left(p,q\right)=1-\frac{1}{N}\frac{2\sum_{x,y,z}\sum_{k}p_{x,y,z}^{k}*q_{x,y,z}^{k}}{\sum_{x,y,z}\sum_{k}p_{x,y,z}^{k}+\sum_{x,y,z}\sum_{k}q_{x,y,z}^{k}}. \end{array}$$

**Figure 9:**
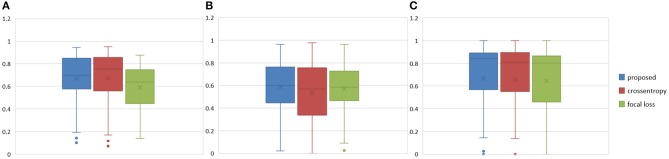
Boxplots for each method in Table 2. Dice similarity scores for **(A)** edemas, **(B)** non-enhancing tumors, and **(C)** enhancing tumors. The symbol “ × ” marks the mean.

**Figure 10:**
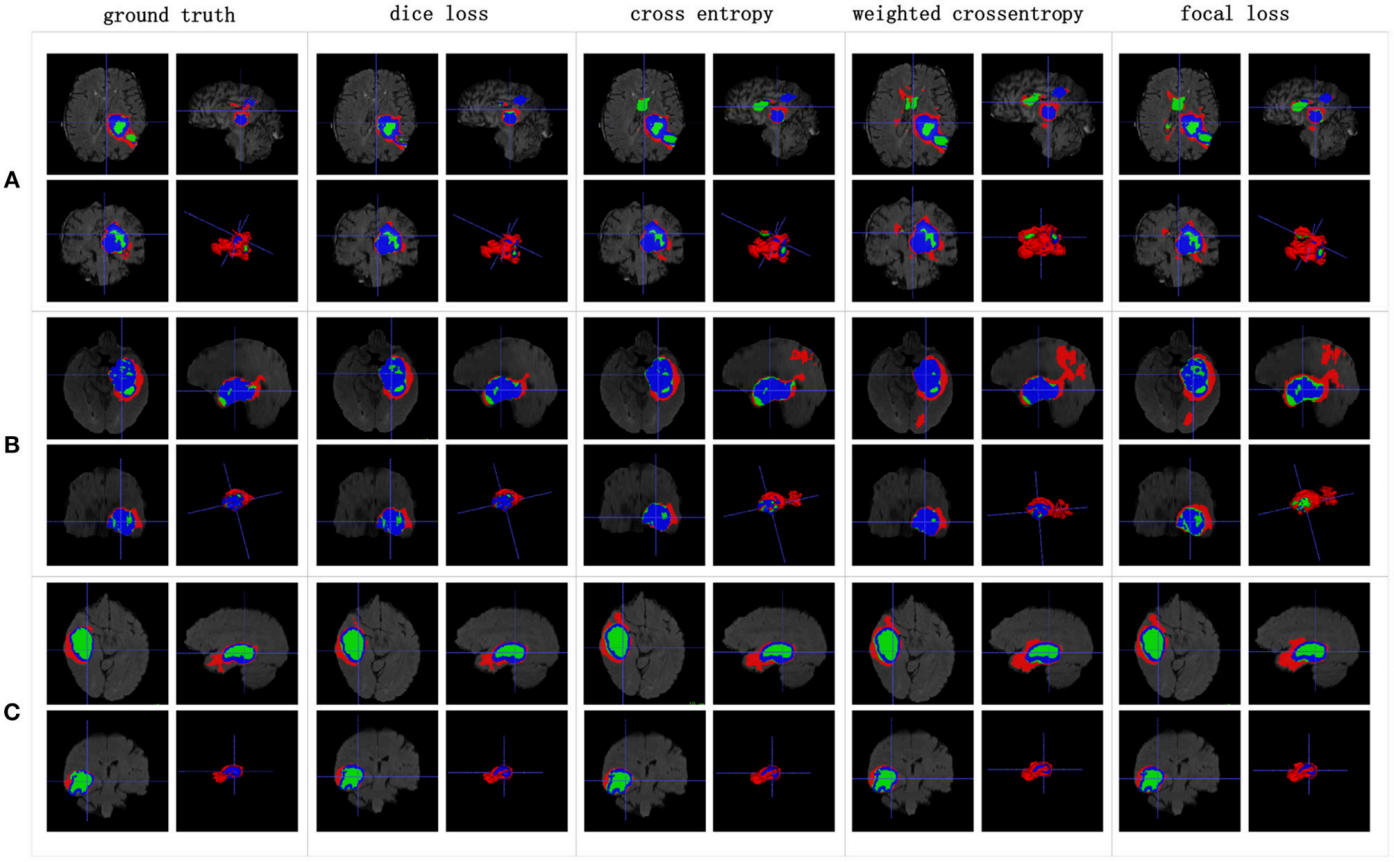
Brain tumor segmentation results predicted by NDNs with different loss functions. The rows represent three samples from different patients, and the columns represent results predicted by NDNs with different losses. Organizers provided ground truth images. **(A-C)** list three samples for different patients.

The Dice similarity scores for the different loss functions used in NDNs are presented in Table 2 and Figure 9. We obtain final scores of 0.6652, 0.5880, and 0.6682 for edemas, non-enhancing tumors, and enhancing tumors, respectively, using the Dice loss. Normal loss functions like the categorical cross-entropy may achieve good results for balanced datasets, but datasets with a massive imbalance among classes require special attention. We avoid weighted categorical cross-entropy as much as possible, because it needs additional hyperparameters that may introduce another difficult problem for network optimization. The results show that the focal loss may be good for binary classification problems to solve intra-class imbalance. However, it is less helpful for inter-class imbalance. The Dice loss is calculated based on the Dice coefficient and can deal with situations with large amounts of class imbalance. Figure 10 shows the ground truth and prediction results for the different loss functions used in NDNs.

**Table 2:** Comparison with different losses: (A) categorical cross-entropy; (B) weighted categorical cross-entropy loss; (C) focal loss; and (D) Dice loss.

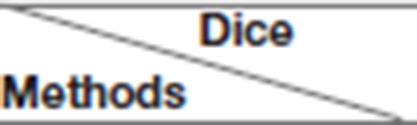	**Edema**	**Non-enhancing tumor**	**Enhancing tumor**
A	**0.6708**	0.5321	0.6579
B	0.6634	0.5774	0.6604
C	0.5905	0.5721	0.6445
D	0.6652	**0.5880**	**0.6682**

*The bold values mean the best result (the highest dice value) for each class*.

### 3.3. Experiment 3

**Figure 11:**
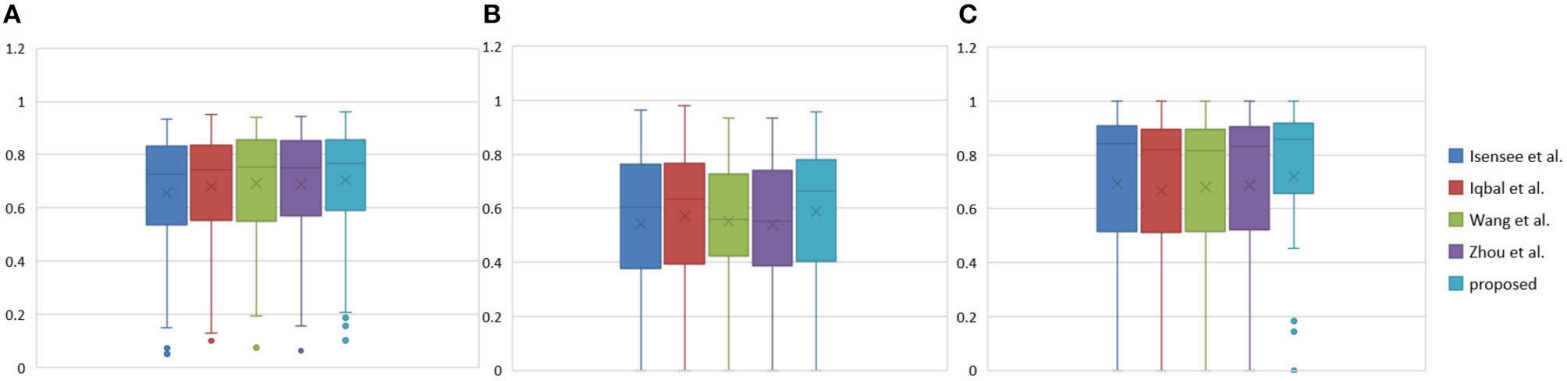
Boxplots for each method in Table 3. Dice similarity scores for **(A)** edemas, **(B)** non-enhancing tumors, and **(C)** enhancing tumors. The symbol “ × ” marks the mean.

We reproduced several state-of-the-art methods for brain tumor segmentation for comparison with our algorithm. Isensee et al. (2017) achieved a high Dice score in the BraTS 2017 Challenge by using a U-Net-like architecture. They employed deep supervision in the localization pathway to integrate segmentation layers at different levels of the network and combined them via element-wise summation to form the final network output. Iqbal et al. (2018) adopted SE blocks at the end of the decoder part and fused its output with the output of encoder blocks. These two methods were chosen for comparison, because they have similarities with our network structure. Wang et al. (2017) proposed a triple-cascaded framework to segment the entire tumor, tumor core, and enhancing tumor core sequentially. They used dilated convolutions after the down-sampling layers and set the dilation parameter from 1 to 3. Zhou et al. (2018) presented a one-single multitask CNN that can learn the correlations between different categories. These two methods used a cascade or cascade-like training strategy like our training process, and they both obtained high Dice scores in the brain tumor segmentation task. In this experiment, a multiclass segmentation problem was decomposed into three binary segmentation problems by repeated training of NDNs with the coarse-to-fine method just like (Wang et al., 2017). Table 3 and Figure 11 present the quantitative evaluation according to the Dice similarity scores for the same datasets.

**Table 3:** Comparison of methods for the same dataset: (A) Isensee et al. (2017); (B) Iqbal et al. (2018); (C) Wang et al. (2017); (D) Zhou et al. (2018); and (E) our proposed method training with the cascade strategy.

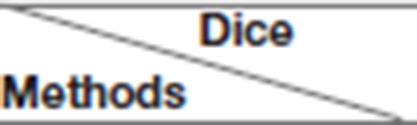	**Edema**	**Non-enhancing tumor**	**Enhancing tumor**
A	0.6574	0.5418	0.6943
B	0.6808	0.5727	0.6661
C	0.6919	0.5504	0.6793
D	0.6894	0.5376	0.6861
E	**0.7043**	**0.5889**	**0.7206**

*The bold values mean the best result (the highest dice value) for each class*.

**Figure 12:**
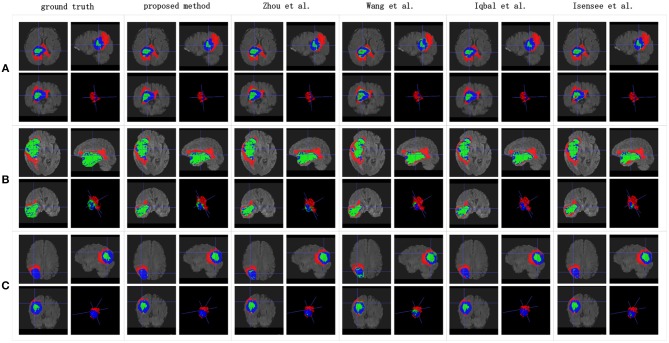
Brain tumor segmentation results predicted by the different algorithms. The rows represent three samples from different patients, and the columns represent algorithms from published papers. Organizers provided the ground truth images. **(A-C)** list three samples for different patients.

Table 3 indicates that the Dice similarity scores of our proposed method are 0.7043, 0.5889, and 0.7206 for edemas, non-enhancing tumors, and enhancing tumors, respectively, which are higher than those of all comparison methods for every class. Moreover, the results are 3.9 and 5.2% higher for edemas and enhancing tumors than when the three classes are trained together, and the results for the non-enhancing tumors do not worsen. These results prove that the cascade training strategy can improve the accuracy for brain tumor segmentation. Figure 12 shows the ground truth and prediction results for different state-of-the-art methods.

## 4. Discussion

### 4.1. Competitive Segmentation Results

U-Net increases the number of up-sampling and skip connections compared with FCN, which can supplement more location information for semantic information. The U-Net architecture has received increasing attention recently and has been shown that it is a stable algorithm for many segmentation tasks. Despite its great success, however, U-Net still has limitations for some specialized tasks.

We found that stacking residual blocks instead of simple convolution layers can improve the brain tumor segmentation performance. This is because residual blocks can fuse receptive fields of different sizes and ease the training of the networks. Attention mechanisms have shown their utility for many computer vision tasks. SE blocks work as an attention mechanism that can explore the relationship between channels to suppress useless information and enhance useful information by fusing global information. They can help a network notice essential features and make correct decisions. Nesting the SE blocks into our base structure causes the corresponding Dice similarity scores of the edemas, non-enhancing tumors, and enhancing tumors to reach 0.6725, 0.536, and 0.638, respectively. To solve the problem of insufficient receptive fields and to simultaneously avoid the gridding issue, we add RnD blocks to the network. By learning from the HDC framework, RnD blocks can enlarge receptive fields by using dilated convolutions with different dilation rates. Based on this, our method obtains results of 0.6652, 0.5880, and 0.6682, respectively.

**Figure 13:**
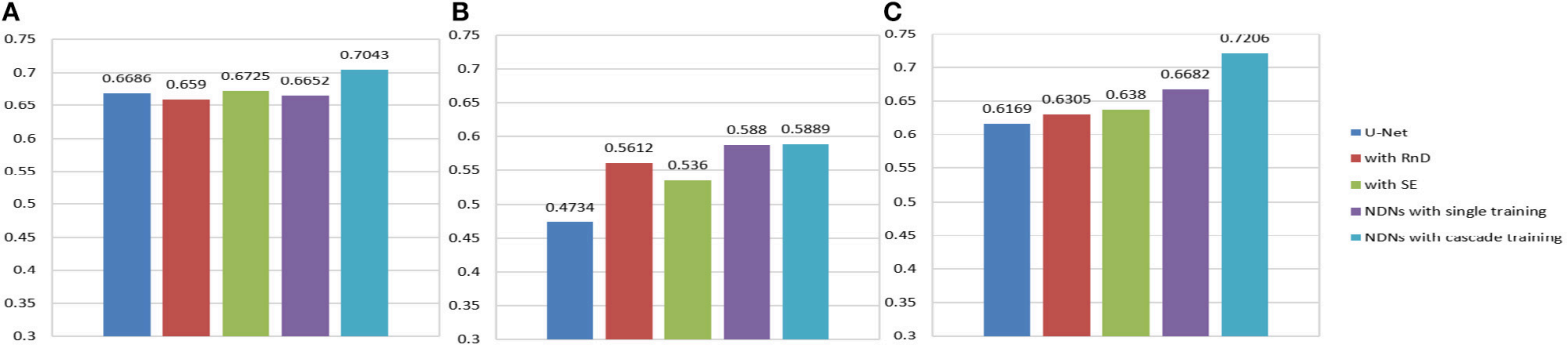
Histogram for each method. Dice similarity scores for **(A)** edemas, **(B)** non-enhancing tumors, and **(C)** enhancing tumors.

**Figure 14:**
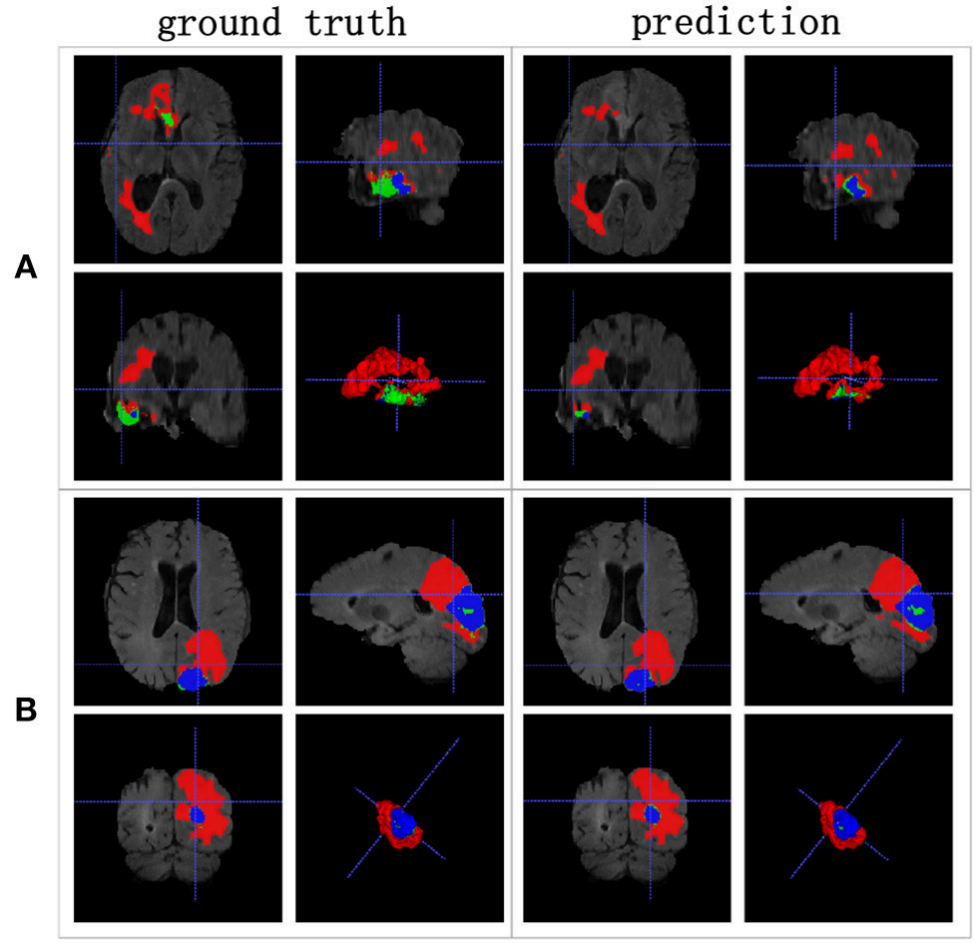
Limitations caused by the class imbalance problem. **(A,B)** present two different samples.

An extreme imbalance between categories affects the segmentation results, especially for edemas, and needs to be addressed. Non-enhancing tumors usually have smaller regions than the other two classes, as shown in **Figure 14**, which will have a negative effect on the segmentation results. In order to alleviate the class imbalance, two measures are taken. First, different losses are employed by NDNs to determine the best performance, and a Dice loss function is eventually selected. Moreover, we borrowed the cascade training strategy adopted by many state-of-the-art methods for brain tumor segmentation. Cascade training can balance the quantitative differences among different classes to some extent. The final results obtained by our proposed method were 0.7043 for edema, 0.5889 for non-enhancing tumors, and 0.7206 for enhancing tumors. The experimental results are shown in Figure 13. These reasonable results are attributed to both the network structure and training strategies.

### 4.2. Limitations

This study is limited by the class imbalance problem, even though some measures have been taken to alleviate it. Some small regions in brain tumors like non-enhancing tumors could not be predicted very well. For example, in the two samples in Figure 14, only 8.5% of the entire tumor is non-enhancing in sample A and 2.25% in sample B. This huge category imbalance lead to inaccurate segmentation results of 0.279 and 0.402 for non-enhancing tumors in samples A and B, respectively. The class imbalance problem remains a challenge that should be addressed in the future.

## 5. Conclusion

Clinical applications of computer-aided systems have gained a great deal of research attention. Supremely accurate brain tumor segmentation is a tedious but vital task for clinicians because of various sizes and shapes of tumors. Quantitative analysis of brain tumors is critical to relieve pressure on doctors and obtain more accurate segmentation results. We developed a new deep learning framework based on U-Net, NDNs, for segmenting brain tumors. Our results showed that NDNs can extract discriminative features of edemas, non-enhancing tumors, and enhancing tumors by obtaining large receptive fields and integrating channel information. Compared with other state-of-the-art methods, NDNs obtained higher Dice similarity scores. The proposed method makes it possible to generate accurate segmentation result for brain tumors without manual interference and provides considerable insight on the application of computer-aided systems to clinical tasks.

## Data Availability

The datasets for this study can be found at Medical Segmentation Decathlon, and the low-grade data can be downloaded from MICCAI BraTS 2018.

## Author's Note

All data are made available online with a permissive copyright-license (CC-BY-SA 4.0), allowing for data to be shared, distributed and improved upon.

## Author Contributions

LW, SW, and RC conceptualized the algorithm design. LW, SW, and SH designed the study. LW, SW, RC, SH, and CL collected the data. LW, SW, SH, and CL analyzed the data. LW, SW, and CL interpreted the data. LW, SW, and SH sourced the literature. LW, SW, RC, and SH wrote the draft. LW, SH, XQ, YC, and CL edited the manuscript. LW and CL acquired the funding and supervised the whole study.

### Conflict of Interest Statement

The authors declare that the research was conducted in the absence of any commercial or financial relationships that could be construed as a potential conflict of interest.
